# Gray matter volume of cerebellum associated with idiopathic normal pressure hydrocephalus: A cross-sectional analysis

**DOI:** 10.3389/fneur.2022.922199

**Published:** 2022-09-07

**Authors:** Minrui Lv, Xiaolin Yang, Xi Zhou, Jiakuan Chen, Haihua Wei, Duanming Du, Hai Lin, Jun Xia

**Affiliations:** ^1^Department of Radiology, Shenzhen Second People's Hospital/The First Affiliated Hospital of Shenzhen University Health Science Center, Shenzhen, China; ^2^Department of Radiology, Southern University of Science and Technology Hospital, Shenzhen, China; ^3^Department of Interventional Therapy, Shenzhen Second People's Hospital/The First Affiliated Hospital of Shenzhen University Health Science Center, Shenzhen, China; ^4^Central Research Institute, United Imaging Healthcare, Shanghai, China

**Keywords:** magnetic resonance imaging, idiopathic normal pressure hydrocephalus, cerebellum, voxel-based morphometry, cerebrospinal fluid

## Abstract

The cause of idiopathic normal pressure hydrocephalus's (iNPH) clinical symptoms remains unclear. The cerebral cortex is the center of the brain and provides a structural basis for complex perception and motor function. This study aimed to explore the relationship between changes in cerebral cortex volume and clinical symptoms in patients with iNPH. This study included 21 iNPH patients and 20 normal aging (NA) controls. Voxel-based morphometry statistical results showed that, compared with NA, the gray matter volumes of patients with iNPH in the bilateral temporal lobe, bilateral hippocampus, bilateral thalamus, bilateral insula, left amygdala, right lenticular nucleus, right putamen, and cerebellum decreased, while the volumes of gray matter in the bilateral paracentral lobules, precuneus, bilateral supplementary motor area, medial side of the left cerebral hemisphere, and median cingulate and paracingulate gyri increased. Correlation analysis among the volumes of white matter and gray matter in the cerebrum and cerebellum and the iNPH grading scale (iNPHGS) revealed that the volume of white matter was negatively correlated with the iNPHGS (*P* < 0.05), while the gray matter volumes of cerebellar area 6 and area 8 were negatively correlated with the clinical symptoms of iNPH (*P* < 0.05). The volume of gray matter in the cerebellar vermis was negatively correlated with gait, and the gray matter volume of cerebellar area 6 was negatively correlated with cognition. Our findings suggest that the cerebellum also plays an important role in the pathogenesis of iNPH, potentially highlighting new research avenues for iNPH.

## Introduction

Idiopathic normal-pressure hydrocephalus (iNPH) is an unexplained disease with gait disorder, cognitive impairment, and urinary incontinence as the main clinical manifestations (1). Cerebrospinal fluid (CSF) shunt surgery is an effective treatment option for iNPH (2, 3).

The imaging manifestations of iNPH have certain characteristics. First, ventricular enlargement can be observed on CT and MRI. The evaluation criteria for ventricular enlargement are the ratio of the maximum distance between the anterior horns of the bilateral ventricles at the same level and the maximum diameter of the skull (Evans' index) ≥0.3 or anterior diameter of the lateral ventricle index (ALVI) >0.5 (4). In addition to ventricular enlargement, iNPH also has the following other brain imaging features: (1) disproportionate enlargement of the subarachnoid space (DESH), which manifests as narrowing of the area above the lateral fissure cisterna, bilateral sulci of the midline, subarachnoid space, and widening of the lateral fissure cisterna and ventricle below it; (2) Angle of the corpus callosum: Connect the anterior joint (AC) to the posterior joint (PC) and measure the angle of the corpus callosum at the coronal plane perpendicular to the AC-PC connection at the posterior joint, wherein a corpus callosum angle of <90° indicates the diagnosis of iNPH; (3) Local sulci expansion: There are single or multiple rounds of oval sulci enlargement near the midline of the brain (2). Although iNPH has more common imaging changes, the imaging manifestations of ventricular enlargement are not specific. The DESH sign and corpus callosum angle are less sensitive in the diagnosis of iNPH (2). In addition, ventricular enlargement and DESH signs cannot explain the cause of the clinical symptoms of iNPH.

The gray matter of the brain, also known as the cortex, is a densely packed part of the neuronal cells, wherein a complex system of connections between the neurons is present. The cerebral cortex is the material basis for brain activity and a high-level center for regulating body functions. The white matter of the brain is where nerve fibers, which transmit nerve impulses, gather. Integrity of the brain structure is a prerequisite for normal brain function. Studies have shown that the degree of brain deformation after an iNPH CSF shunt is related to preoperative clinical symptoms (5, 6). Voxel-based morphometry (VBM) is a comprehensive and objective brain structure analysis method that replaces the traditional method of manually delineating a region of interest (ROI) and improves the accuracy and repeatability of measurements. VBM can be used to analyze brain magnetic resonance images at the voxel level; calculate the density and volume of brain gray matter, white matter, and CSF; and accurately display brain morphological changes (7). Previous studies on the morphological changes in iNPH have focused on the brain. Ishii et al. (8) used VBM8 to analyze the changes in gray matter density of iNPH and found that the insular lobe, caudate, and thalamus gray matter density of patients with iNPH decreased significantly. A study on changes in the thickness of the iNPH cortex by Kang et al. (9) showed that the cerebral cortex adjacent to the longitudinal fissure thickened, and the gait of iNPH was negatively correlated with the thickness of the superior frontal gyrus, straight gyrus, superior temporal gyrus, temporal pole, and medial orbital cortex of the insula. The traditional view is that the cerebellum maintains balance and regulates muscle tension, which means that cerebellar injuries can cause ataxia. Many studies have shown that the cerebellum is an important structure for urination control (8, 10) and plays an important role in many cognitive fields (11, 12). The function of the cerebellum overlaps with the clinical manifestations of iNPH, but there is no research on the characteristic changes in iNPH cerebellar morphology.

The purpose of this study was to use CAT12 to obtain objective and fine measurements of iNPH high-resolution brain structure images, to identify morphological changes in the cerebrum and cerebellum of patients with iNPH, and to explore the correlation between morphological changes and clinical symptoms. We speculate that the white matter volume change in patients with iNPH and the characteristic gray matter volume change pattern in patients with iNPH are related to the clinical symptoms of iNPH.

## Materials and methods

### Research objects

The subjects of the study were retrospectively enrolled patients who were clinically diagnosed with definite iNPH at Shenzhen Second People's Hospital from September 2018 to November 2020. The inclusion criteria for definite iNPH were in accordance with the guidelines (13). High-resolution T1WI cranial MRI scans were collected before CSF shunt surgery. The exclusion criteria were as follows: (1) patients with a history of other neurological diseases such as a tumor, cerebrovascular disease, brain trauma, or mental illness; (2) patients with poor image quality, which is not suitable for the study. The iNPH grading scale (iNPHGS) (14) of patients with iNPH before CSF shunt placement, along with the age, sex, blood pressure, and intracranial pressure were collected. In addition, a normal aging control group (NA) matched for age and sex were included. All participants were right-handed and of Han nationality. The NA group underwent MRI examination only, and their age, sex, and blood pressure were recorded. The protocol was approved by our Hospital Bioethics Committee (approval no. KS20190114001).

### INPHGS evaluation

The iNPHGS was used to score the clinical system (14). The iNPHGS includes the evaluation of three clinical symptoms: cognition, gait, and urinary control. The score for each clinical symptom ranged from 0 to 4 and the total score ranged from 0 to 12. The higher the score, the more severe the symptom. Cognitive-iNPHGS (r-iNPHGS), gait-iNPHGS (g-iNPHGS), and urinary control-iNPHGS (u-iNPHGS) were used for independent evaluation of symptoms of cognition, gait, and urinary control, respectively.

### MRI scan parameters

All sequences were obtained using a 3.0T MRI scanner (Prisma, Siemens, Erlangen, Germany). Scanning adopted 64-channel head matrix coils and high-resolution T1WI. The image parameters were as follows: TR = 8.656 ms, TE = 3.22 ms, inversion time (TI) = 450 ms, FOV = 256 × 256, slice thickness = 1 mm, voxel size = 1 × 1 × 1 mm^3^.

### Intracranial pressure measurement

Intracranial pressure was measured by experienced neurosurgeons. The patient was placed in a lateral position, and a lumbar puncture was performed between the 3^rd^ and 4^th^ lumbar intervertebral spaces. Blood pressure in the supine position was measured before the lumbar puncture.

### Image processing and statistical analysis

The T1-weighted structural images were processed and analyzed using the CAT12 toolbox (http://dbm.neuro.uni-jena.de/cat/) implemented in SPM12 (http://www.fil.ion.ucl.ac.uk/spm/software/spm12/). On T2-weighted images, each iNPH subject's white matter hyperintensities were segmented using ITK-SNAP (http://www.itksnap.org). Additionally, the volume of brain white matter was corrected using the segmented hyperintensities mask (15). The main steps included spatial normalization, segmentation, estimating total intracranial volume (TIV), checking sample homogeneity, and smoothing, with default settings unless indicated otherwise. The normalized gray matter maps measures were smoothed with 8-mm FWHM Gaussian kernels. The AAL116 template (Supplementary document) was used to divide the gray matter structure, which included the gray matter regions of the cerebrum and cerebellum, covering 90 regions of the cerebrum (45 regions of the left and right cerebrums) and 26 regions of the cerebellum (16). Two-tailed *t*-tests were used to compare gray matter volume (VBM) between patients and healthy controls using age, sex, and TIV as covariates. After GRF correction for multiple comparisons, voxel *P*-value < 0.001 was deemed statistically significant. BrainNet viewer (https://www.nitrc.org/projects/bnv) was used to visualize the statistical results.

Spearman and Pearson correlation analysis was used to analyze total brain gray matter, total brain white matter, and total CSF, and their relationship to clinical symptoms, *P*-value < 0.05 (FDR corrected). We focused on regions of the gray matter cortex that were statistically significant in the VBM analysis to evaluate the connection between gray matter volume alterations and clinical symptoms in iNPH patients. *P*-value < 0.05 (FDR corrected) in Pearson or Spearman correlation analysis are considered statistically significant. The statistics of demographic data and correlation analysis were carried out using EmpowerStats statistical software (http://www.empowerstats.com) and statistical software package R (version 3.2.3), wherein the results of the demographic data were expressed as mean ± SD.

## Results

### Demographic and baseline data

**Figure 1:**
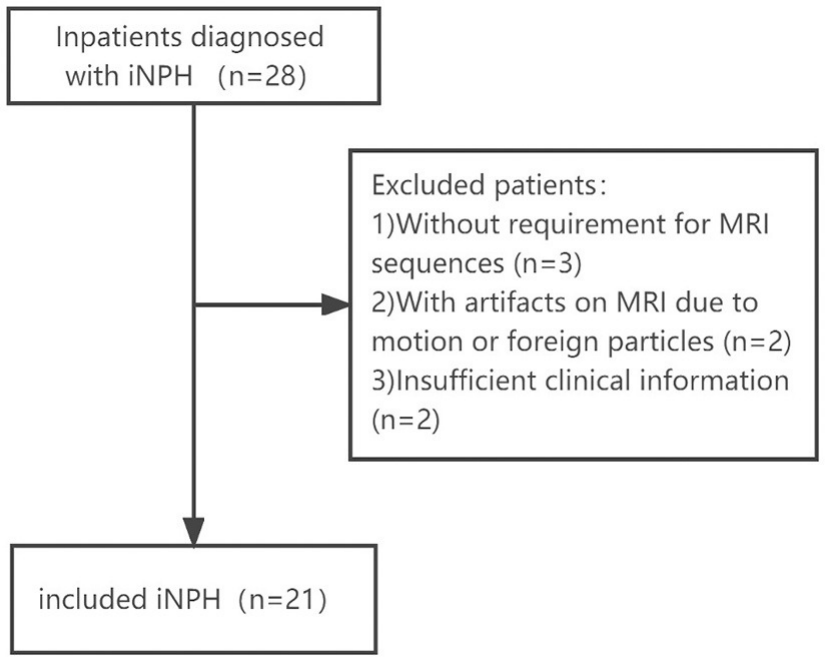
Flowchart of enrollment for patients with idiopathic normal pressure hydrocephalus.

The study includes 21 patients with iNPH (9 females and 12 males) with an average age of 68.04 ± 9.99 years and 20 cases in the NA control group (10 females and 10 males) with an average age of 66.77 ± 4.91 years (Figure 1). There were no significant differences (*P* > 0.05) in age, sex, systolic blood pressure, or diastolic blood pressure between the two groups. The intracranial pressure in the iNPH group was 136 ± 32.09 mmH_2_O. Table 1 provides the descriptive statistics of the basic characteristics of the study population.

**Table 1:** Demographics of the iNPH and NA groups.

**Group**	**NA (*n* = 20)**	**iNPH (*n* = 21)**	***P*-value**
Age (years)	66.77 (4.91)	68.04 (9.99)	0.594
Gender (Male: Female)	10:10	12:9	0.463
Systolic blood pressure (mm Hg)	129.59 (20.32)	125.47 (17.43)	0.658
Diastolic blood pressure (mm Hg)	77.77 (10.32)	78.95 (11.93)	0.724
Intracranial pressure (mmH_2_0)	/	136.32 (32.10)	/
iNPHGS	/	4.81 (2.58)	/
mRS	/	2.619 ± 0.865^*^	/

* Mean + SD / N (%). iNPHGS, idiopathic normal pressure grade scale; mRS, modified Rankin Scale; NA, normal aging.

### Volume changes of INPH total brain gray matter, total brain white matter, total CSF and their relationships with clinical symptoms

**Figure 2:**
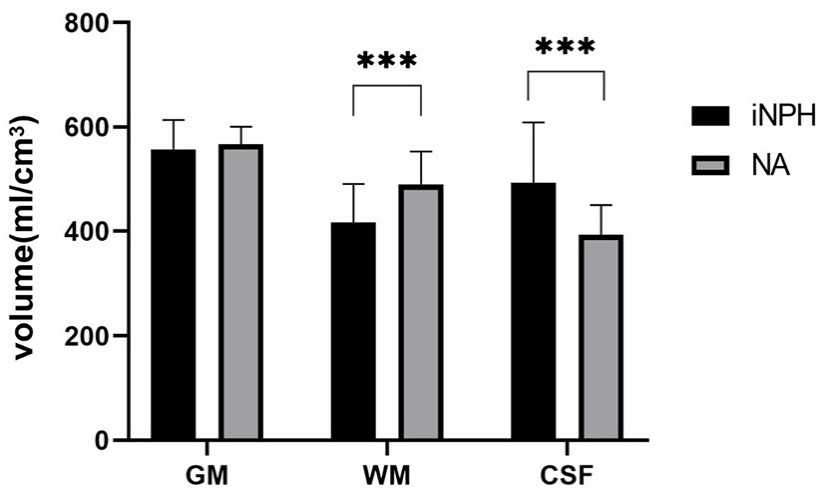
The difference in gray matter volume between the idiopathic normal-pressure hydrocephalus (iNPH) group and normal aging (NA) group. iNPH, idiopathic normal-pressure hydrocephalus; NA, normal aging; GM, gray matter; WM, White matter; CSF, Cerebrospinal fluid. ***P ≤ 0.001.

The results of the two-sample *t*-test (Figure 2) shows that the total brain gray matter volume of the iNPH group is 557.10 ± 55.89 cm^3^ and the total brain gray matter volume of the NA group is 566.84 ± 33.74 cm^3^. There was no significant difference in the total brain gray matter volume between the two groups (*P* = 0.506). The volume of white matter in the NA group was 417.52 ± 73.09 cm^3^, which was significantly lower than that in the NA group (490.34 ± 62.31 cm^3^) (*P* = 0.001). The CSF volume of the iNPH group was 492.75 ± 115.74 cm^3^, which was significantly higher than the volume of 393.67 ± 56.65 cm^3^ in the NA group (*P* = 0.001). The correlation analysis results showed that the white matter volume of iNPH was negatively correlated with iNPHGS (r = −0.4496; *P* = 0.0409), and the total brain gray matter (*P* = 0.3861) and CSF volume (*P* = 0.192) of iNPH were not significantly correlated with iNPHGS.

### Comparison of the gray matter volume in 116 regions of the whole brain between INPH and NA

**Figure 3:**
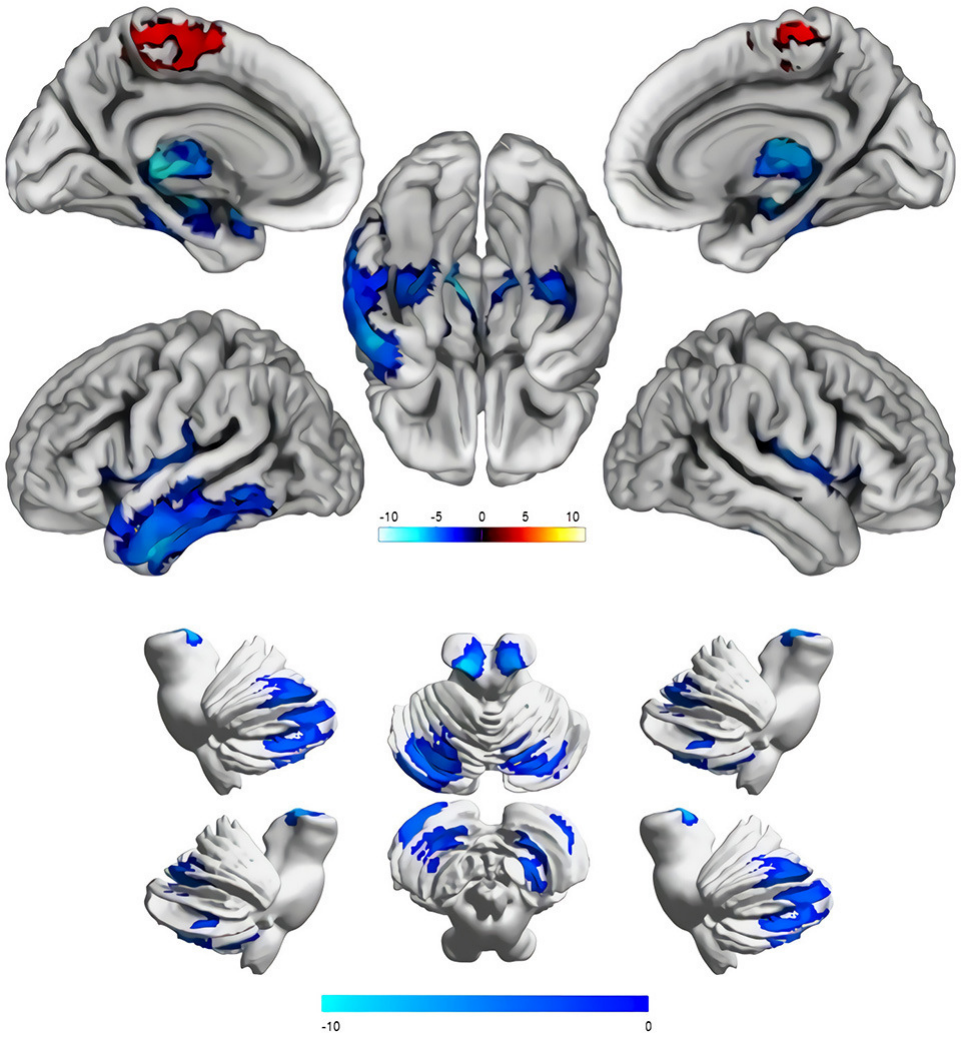
Comparison results of the cerebral and cerebellar gray matter volume between the idiopathic normal-pressure hydrocephalus group and normal aging group (Cluster *P* < 0.05, Voxel *P* < 0.001, GRF corrected). Blue areas indicate reduced gray matter volume, and the red areas indicate increased gray matter volume. The color shade of the color bar represents the size of the T value.

VBM (CAT12) was used to compare gray matter volumes between the two groups (cluster *P* < 0.05, voxel *P* < 0.001, GRF corrected), which showed that there were significant differences in a total of seven gray matter clusters (Table 2). The gray matter volume of iNPH was smaller than that of NA in six clusters, two of which were located in the cerebellum, whereas the other four were located in the cerebrum. In the frontal-parietal lobe of the cerebrum, the gray matter volume of one cluster of iNPH increased. The results showed that compared with NA, the gray matter volumes of iNPH in the bilateral temporal lobe, bilateral hippocampus, bilateral thalamus, bilateral insula, left amygdala, right lenticular nucleus, right putamen (PUT.R), and cerebellum (bilateral anterior cerebellar lobes, bilateral posterior cerebellar lobes, bilateral cerebellar feet, cerebellar vermis) decreased, while the volumes of gray matter in the bilateral paracentral lobules, precuneus, bilateral supplementary motor area, medial side of the left cerebral hemisphere, and median cingulate and paracingulate gyri increased (Figure 3; Table 2).

**Table 2:** Differences between clusters of the iNPH and NA groups.

	**Cluster**	**Regions (AAL)**	**Peak intensity (T)**	**Peak MNI coordinates**			**Cluster size**
				**x**	**y**	**z**	
Volume reduction	Cluster 1	lCbeCru1 // lCbe6 // rCbe6 // lCbeCru2 // rCbeCru1 // lCbe8 // lCbe7b // lCbe4-5 // rCbe4-5 //rCbeCru2 // Ver4-5 // Ver7 // Ver6	−6.13775	13.5	−67.5	−24	6,172
	Cluster 2	THA.L // THA.R // HIP.L // HIP.R // FFG.L // FFG.R // PHG.L // PHG.R //ITG.L	−10.4764	−31.5	−22.5	−12	6,132
	Cluster 3	MTG.L // ITG.L TPOsup.L // TPOmid.L	−6.53961	−51	−3	−34.5	5,496
	Cluster 4	ROL.R // INS.R // STG.R // POsup.R // HES.R //PUT.R	−5.39656	39	−13.5	13.5	2,229
	Cluster 5	INS.L // ROL.L // PoCG.L // STG.L // HES.L // IFGoperc.L	−5.34766	−42	4.5	3	2,175
	Cluster 6	rCbe8	−4.35564	22.5	−61.5	−55.5	1,030
Volume increase	Cluster 7	SMA.L // SMA.R // PCL.L // PCL.R // PCUN.L // DCG.L	4.72536	3	−27	72	1,293

iNPHGS, idiopathic normal pressure grade scale; NA, normal aging.

### Correlation analysis between gray matter volume and clinical symptoms of INPH

**Figure 4:**
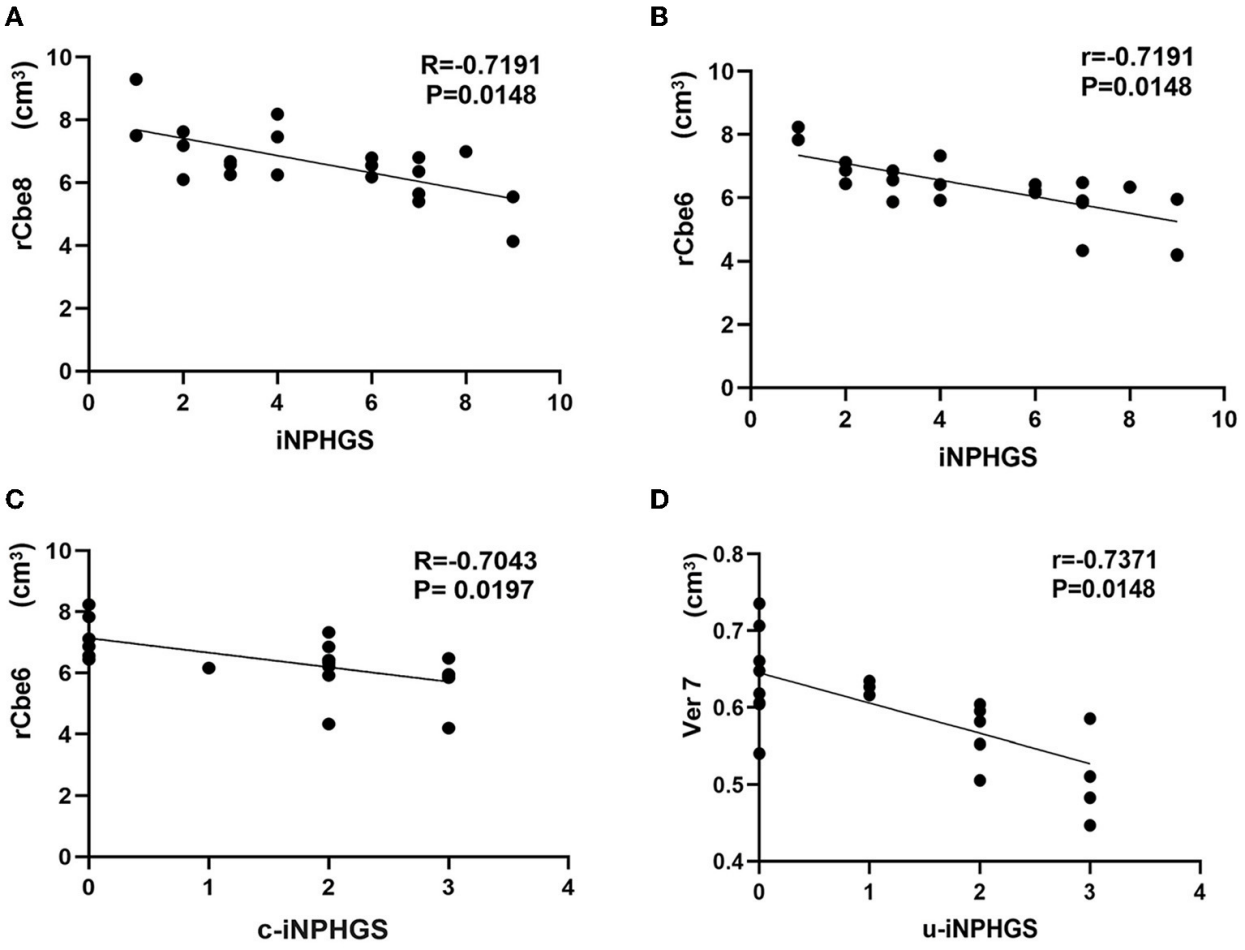
Correlation analysis between different brain regions and the idiopathic normal-pressure hydrocephalus grading scale (iNPHGS). c-iNPHGS, cognitive-iNPHGS; u-iNPHGS, urinary control-iNPHGS; Cerebelum-8-R (rCbe8), Cerebelum-6-R (rCbe6), Vermis-7 (Ver7).

Correlation analysis between different brain regions and iNPHGS shows that rCbe8 and rCbe6 were significantly negatively correlated with iNPHGS (Figures 4A,B). rCbe6 was negatively correlated with r-iNPHGS (Figure 4C). Ver7 expression was negatively correlated with u-iNPHGS (Figure 4D).

## Discussion

Our study found that the total brain white matter volume of patients with iNPH was significantly lower than that of the NA group. The CSF volume was also significantly greater in the iNPH group than that of the NA group, and there was no significant difference in the total brain gray matter volume between the two groups. Both Ishii et al. [2018] and Miskin et al. (17) studies on the morphology of the iNPH brain found that the volume of CSF increased significantly. Miskin et al. (17) also found that the total gray matter volume of the iNPH and healthy control groups did not differ significantly. The results of this study are consistent with these findings. This study also found that the decrease in total white matter volume may be related to the deformation caused by mechanical compression of ventricular expansion. In addition, the volume of the white matter of the brain was negatively correlated with the total score of the iNPHGS, suggesting that the more obvious the deformation of the white matter caused by pressure, the more severe the clinical symptoms. A study on iNPH using diffusion tensor imaging (DTI) found changes in the microstructure of the white matter of the brain, damage to the white matter axons around the ventricle, and gliosis (18). A DTI study also found corticospinal tract injury in patients with iNPH (19). The corticospinal tract neurons are conduction bundles that control the voluntary movement of skeletal muscles, located in the precentral motor area of the cerebral cortex, and terminate the spinal forefoot motor neurons. Therefore, corticospinal tract injuries may be the cause of gait disorders. Using arterial spin-labeling perfusion (ASL) imaging, Virhammar et al. (20) found a reduction in white matter perfusion around the ventricles, and that the reduction of iNPH white matter perfusion is correlated with impaired cognitive function. However, ASL cannot predict the prognosis after shunting (21). Lin et al. (22) and Fattahi et al. (23) used elastography to show that the stiffness of the white matter around the iNPH ventricle is significantly reduced, which also shows that the white matter microstructure of iNPH is damaged. In this study, the quantitative calculation of iNPH white matter volume showed that an enlarged ventricle and a decrease in the volume of white matter under pressure are related to clinical symptoms.

Using the AAL116 template, the cerebrum was divided into 90 gray matter regions (45 in cerebrum and 45 in cerebrum), and the cerebellum was divided into 20 regions. Comparing the 116 regions between groups, the results showed that the gray matter volumes of patients with iNPH in the bilateral temporal lobe, bilateral hippocampus, bilateral thalamus, bilateral insula, left amygdala, right lenticular nucleus, and PUT.R in the cerebrum decreased, while the volumes of gray matter in the bilateral frontal-parietal lobe adjacent to the longitudinal fissure (bilateral paracentral lobules, precuneus, bilateral supplementary motor area, medial side of the left cerebral hemisphere, and median cingulate and paracingulate gyri) increased. Gray matter volumes of multiple regions in the cerebellum (Bilateral anterior cerebellar lobes, bilateral posterior cerebellar lobes, bilateral cerebellar feet, cerebellar vermis) decreased. Our results are roughly the same as those of previous studies, which found that gray matter volumes of the temporal lobe, hippocampus, and thalamus decreased and volumes of the cerebral frontal-parietal lobe adjacent to the longitudinal increased, which is consistent with the diagnosis signs of DESH by iNPH imaging. However, the previous study did not focus on the relationship between clinical symptoms and gray matter volume reduction. This study is the first to explore changes in cerebellar gray matter volume in iNPH and found that cerebellar volume changes are significantly related to clinical symptoms.

This study found that the cerebellar volume of iNPH patients is reduced and that the cerebellar volume of iNPH is negatively correlated with clinical symptoms. Several previous studies on iNPH have also observed abnormal changes in the cerebellum. For example, Virhammar et al. (20) found that the reduction in cerebellar perfusion in patients with iNPH is related to cognitive symptoms through ASL technology. Using elastography, Perry et al. (24) found that urinary incontinence in patients with iNPH is related to an abnormal increase in stiffness of the cerebellum, cerebrum, and frontal lobe. These studies have shown that abnormal changes in the cerebellum are closely related to iNPH symptoms.

Many neuroscientists have explored the functions of the cerebellum in many aspects, indicating that the cerebellum plays an important role in gait control, urination control, and participation in cognitive processes. In terms of cognition, previous studies on neuroanatomy, clinical practice, and neuroimaging have shown that the cerebellum is involved in the process of cognitive tasks. The cerebellum is involved in the activation of emotions, executive functions, language, music, working memory, and other cognitive fields (11, 25). This study found that cerebellar area 6 in patients' iNPH was negatively correlated with cognitive function. Based on our results, it can be inferred that iNPH cognitive impairment is related to the inhibition of cerebellar area 6 function. In terms of urination control, the cerebellum (especially the cerebellar vermis) has a strong inhibitory effect on the urination reflex and promotes the bladder emptying process (26, 27). A study, conducted on an acute encephalitis patient with ataxia and urinary dysfunction after cerebellar atrophy, found decreased cerebellar perfusion on SPECT and detrusor overactivity on urodynamic examination. These findings indicate that degenerative changes in the cerebellar vermis are one of the causes of urinary dysfunction (28). In addition, a study on the mechanism of iNPH bladder dysfunction by Japanese scientists Sakakibara et al. (29) found that the symptoms of iNPH urinary frequency, urgency, and incontinence are caused by excessive detrusor muscle activity. Therefore, based on our research results, it is inferred that the cause of urinary control disorder in patients with iNPH is that ventricular expansion leads to compression of the cerebellum, especially the cerebellar vermis, which weakens the inhibitory effect of the cerebellum on the micturition reflex, and excessive contraction of the detrusor leads to urinary incontinence. In terms of motor control, Coffman et al. (30) explored the function of the cerebellar vermis and found that many neurons in the primary motor cortex of the inner hemisphere and several motor areas project to the cerebellar vermis. Neural circuits in the cerebral cortex and cerebellar vermis are involved in the regulation of body posture and movements. The cerebellar vermis is the projection area of the motor cortex, and it is speculated that the cerebrum-cerebellar vermis-related neural circuit disorder is the basis of some forms of muscle dysfunction, which may also cause volume changes in the cerebellar vermis of the iNPH patient causing gait disorders. A study on the coordinated movement of mice found that the cerebellar vermis plays an important role in controlling the speed and direction of movement (31). Studies have found that patients with cerebellar vermis infarction have symptoms of gait ataxia (32). These studies have shown that the cerebellar vermis plays an important role in gait control; however, our study found that the correlation between iNPH cerebellar vermis volume changes and gait disorder score u-iNPHGS was not significant (r = −0.5312; *P* = 0.11). The effect of cerebellar gray matter structure changes on gait in iNPH requires further study.

At present, research studies on iNPH mostly focus on brain morphological changes, such as changes in ventricular morphology, differences in CSF distribution, evaluation and application of the Evans' index, corpus callosum angle, and DESH sign (2). However, these studies could not explain these symptoms. An increasing number of studies have found that the cerebellum plays an important role in the pathophysiology, occurrence, and development of neuropsychiatric diseases. However, research on cerebellar changes in iNPH is limited. This study is the first to explore the changes in cerebellar volume in iNPH. Our research found that a decrease in cerebellar volume is closely related to the clinical symptoms of iNPH. Therefore, we believe that changes in the cerebellum should not be ignored when studying iNPH. Our innovative research shows that the clinical symptoms of iNPH are not affected solely by the cerebrum but are the result of damage to the overall cerebrum-cerebellum function. We speculate that this may be related to abnormal changes in the cerebrum-cerebellar neural circuit. In addition, the segmentation of gray matter, white matter, and CSF in this study used a voxel-based brain morphology research tool named CAT12 (http://dbm.neuro.uni-jena.de/cat/). Previous studies have used the VBM8 (http://dbm.neuro.uni-jena.de/vbm8/) tool to study iNPH. Multiple studies have shown that CAT12 can provide a more accurate brain morphology analysis than VBM8 and can detect subtle changes in brain structure (33, 34).

This study has certain limitations. First, the patients with iNPH included were clinically suspected patients with iNPH, but the prognosis of iNPH shunt is different, which may be caused by the different degrees of damage to the brain and cerebellum microstructure due to the duration of the disease. Early identification of patients with suspected iNPH also has significance. In addition, the study is a cross-sectional study, which cannot explain the reasons for the improvement of patients' symptoms after a CSF shunt. Studies have shown that the periaqueductal gray (PAG) is an important structure for the control of urination (33, 34). Studies have speculated that the microstructural changes of PAG are related to the symptoms of lower urinary tract dysfunction in iNPH (35, 36), but the AAL116 gray matter template used in this study does not include PAG. Exploration of PAG is a direction for further research.

The clinical symptoms of iNPH lack specificity and are easily misdiagnosed as Alzheimer's disease (AD) or Parkinson's syndrome. The cause of cognitive dysfunction in AD is usually attributed to the atrophy of the temporal lobe and hippocampus, as well as to a certain degree of cerebellar atrophy (37). Atrophy of the frontal lobe and cerebellum (38) is observed in early Parkinson's disease with mild cognitive impairment. The treatment methods for these three diseases are different. Early detection and timely intervention can delay the development of the disease and improve prognosis. Through this study, we speculate that the pattern of gray matter volume changes in the cerebrum and cerebellum is helpful in the diagnosis of iNPH, AD, Parkinson's syndrome, and other diseases. In addition, the relationship between the morphological changes of the cerebrum and cerebellum before and after iNPH CSF shunt, and the improvement of clinical symptoms remains to be further studied.

## Conclusion and perspective

This study explored the relationship between the morphological changes in the cerebrum and cerebellum, and the clinical symptoms of patients with iNPH. The decrease in total white matter volume of iNPH was negatively correlated with the iNPHGS. It was also found that a decrease in the gray matter volume of the iNPH cerebellum is negatively associated with clinical symptoms of iNPH. This study innovatively shows that the clinical symptoms of iNPH are not solely affected by the cerebrum, but also by the cerebrum-cerebellum interaction. The cerebellar also plays an important role in the pathogenesis of iNPH. Future research should focus on the relationship between clinical symptoms and overall changes in the cerebrum and cerebellum of patients with iNPH. For example, using fMRI and DTI in whole-brain functional network analysis to investigate the topology of the brain of iNPH patients, this may provide a comprehensive understanding of the pathophysiology of iNPH.

## Data availability statement

The raw data supporting the conclusions of this article will be made available by the authors, without undue reservation.

## Ethics statement

The studies involving human participants were reviewed and approved by Shenzhen Second People's Hospital Bioethics Committee (Approval No. KS20190114001). The patients/participants provided their written informed consent to participate in this study. Written informed consent was obtained from the individual(s) for the publication of any potentially identifiable images or data included in this article.

## Author contributions

ML designed and conducted most of the experiments and drafted the manuscript. XY, JC, XZ, DD, HL, and HW helped with design and data collection. JX supervised this project. All authors contributed to the article and approved the submitted version.

## Funding

This work was supported in part by the National Natural Science Foundation of China (Grant Number: 82171913) and the Shenzhen Key Medical Discipline Construction Fund (Grant Number: SZXK052).

## Conflict of interest

The authors declare that the research was conducted in the absence of any commercial or financial relationships that could be construed as a potential conflict of interest.

## Publisher's note

All claims expressed in this article are solely those of the authors and do not necessarily represent those of their affiliated organizations, or those of the publisher, the editors and the reviewers. Any product that may be evaluated in this article, or claim that may be made by its manufacturer, is not guaranteed or endorsed by the publisher.
