# Supplementary material for: Gray matter volume of cerebellum associated with idiopathic normal pressure hydrocephalus: A cross-sectional analysis
Source: Front Neurol. 2022 Sep 7;13:922199. doi: 10.3389/fneur.2022.922199 (PMC9489844; doi:10.3389/fneur.2022.922199)
Supplement: Supplementary file 1 [file Presentation_1.pdf]

Anatomical Automatic Labeling

Labels	Mricro Regions	Regions	abbreviation	Anatomical classification
1	Amygdala_L	Amygdala	AMYG.L	Temporal
2	Amygdala_R	Amygdala	AMYG.R	Temporal
3	Angular_L	Angular gyrus	ANG.L	Parietal
4	Angular_R	Angular gyrus	ANG.R	Parietal
5	Calcarine_L	Calcarine fissure and surrounding cortex	CAL.L	Occipital
6	Calcarine_R	Calcarine fissure and surrounding cortex	CAL.R	Occipital
7	Caudate_L	Caudate nucleus	CAU.L	Subcortical
8	Caudate_R	Caudate nucleus	CAU.R	Subcortical
9	Cingulum_Ant_L	Anterior cingulate and paracingulate gyri	ACG.L	Prefontal
10	Cingulum_Ant_R	Anterior cingulate and paracingulate gyri	ACG.R	Prefontal
11	Cingulum_Mid_L	Median cingulate and paracingulate gyri	DCG.L	Frontal
12	Cingulum_Mid_R	Median cingulate and paracingulate gyri	DCG.R	Frontal
13	Cingulum_Post_L	Posterior cingulate gyrus	PCG.L	Parietal
14	Cingulum_Post_R	Posterior cingulate gyrus	PCG.R	Parietal
15	Cuneus_L	Cuneus	CUN.L	Occipital
16	Cuneus_R	Cuneus	CUN.R	Occipital
17	Frontal_Inf_Oper_L	Inferior frontal gyrus, opercular part	IFGoperc.L	Prefontal
18	Frontal_Inf_Oper_R	Inferior frontal gyrus, opercular part	IFGoperc.R	Prefontal
19	Frontal_Inf_Orb_L	Inferior frontal gyrus, orbital part	ORBinf.L	Prefontal
20	Frontal_Inf_Orb_R	Inferior frontal gyrus, orbital part	ORBinf.R	Prefontal
21	Frontal_Inf_Tri_L	Inferior frontal gyrus, triangular part	IFGtriang.L	Prefontal
22	Frontal_Inf_Tri_R	Inferior frontal gyrus, triangular part	IFGtriang.R	Prefontal
23	Frontal_Mid_Orb_L	Middle frontal gyrus, orbital part	ORBmid.L	Prefontal
24	Frontal_Mid_Orb_R	Middle frontal gyrus, orbital part	ORBmid.R	Prefontal
25	Frontal_Mid_L	Middle frontal gyrus	MFG.L	Prefontal
26	Frontal_Mid_Orb_L	Superior frontal gyrus, medial orbital	ORBsupmed.L	Prefontal
27	Frontal_Mid_Orb_R	Superior frontal gyrus, medial orbital	ORBsupmed.R	Prefontal
28	Frontal_Mid_R	Middle frontal gyrus	MFG.R	Prefontal
29	Frontal_Sup_L	Superior frontal gyrus, dorsolateral	SFGdor.L	Prefontal
30	Frontal_Sup_Medial_L	Superior frontal gyrus, medial	SFGmed.L	Prefontal
31	Frontal_Sup_Medial_R	Superior frontal gyrus, medial	SFGmed.R	Prefontal
32	Frontal_Sup_Orb_L	Superior frontal gyrus, orbital part	ORBsup.L	Prefontal
33	Frontal_Sup_Orb_R	Superior frontal gyrus, orbital part	ORBsup.R	Prefontal
34	Frontal_Sup_R	Superior frontal gyrus, dorsolateral	SFGdor.R	Prefontal
35	Fusiform_L	Fusiform gyrus	FFG.L	Temporal

36	Fusiform_R	Fusiform gyrus	FFG.R	Temporal
37	Heschl_L	Heschl gyrus	HES.L	Temporal
38	Heschl_R	Heschl gyrus	HES.R	Temporal
39	Hippocampus_L	Hippocampus	HIP.L	Temporal
40	Hippocampus_R	Hippocampus	HIP.R	Temporal
41	Insula_L	Insula	INS.L	Subcortical
42	Insula_R	Insula	INS.R	Subcortical
43	Lingual_L	Lingual gyrus	LING.L	Occipital
44	Lingual_R	Lingual gyrus	LING.R	Occipital
45	Occipital_Inf_L	Inferior occipital gyrus	IOG.L	Occipital
46	Occipital_Inf_R	Inferior occipital gyrus	IOG.R	Occipital
47	Occipital_Mid_L	Middle occipital gyrus	MOG.L	Occipital
48	Occipital_Mid_R	Middle occipital gyrus	MOG.R	Occipital
49	Occipital_Sup_L	Superior occipital gyrus	SOG.L	Occipital
50	Occipital_Sup_R	Superior occipital gyrus	SOG.R	Occipital
51	Olfactory_L	Olfactory cortex	OLF.L	Prefontal
52	Olfactory_R	Olfactory cortex	OLF.R	Prefontal
53	Pallidum_L	Lenticular nucleus, pallidum	PAL.L	Subcortical
54	Pallidum_R	Lenticular nucleus, pallidum	PAL.R	Subcortical
55	Paracentral_Lobule_L	Paracentral lobule	PCL.L	Parietal
56	Paracentral_Lobule_R	Paracentral lobule	PCL.R	Parietal
57	ParaHippocampal_L	Parahippocampal gyrus	PHG.L	Temporal
58	ParaHippocampal_R	Parahippocampal gyrus	PHG.R	Temporal
59	Parietal_Inf_L	Inferior parietal, but supramarginal and angular gyri	IPL.L	Parietal
60	Parietal_Inf_R	Inferior parietal, but supramarginal and angular gyri	IPL.R	Parietal
61	Parietal_Sup_L	Superior parietal gyrus	SPG.L	Parietal
62	Parietal_Sup_R	Superior parietal gyrus	SPG.R	Parietal
63	Postcentral_L	Postcentral gyrus	PoCG.L	Parietal
64	Postcentral_R	Postcentral gyrus	PoCG.R	Parietal
65	Precentral_L	Precental gyrus	PreCG.L	Frontal
66	Precentral_R	Precental gyrus	PreCG.R	Frontal
67	Precuneus_L	Precuneus	PCUN.L	Parietal
68	Precuneus_R	Precuneus	PCUN.R	Parietal
69	Putamen_L	Lenticular nucleus, putamen	PUT.L	Subcortical
70	Putamen_R	Lenticular nucleus, putamen	PUT.R	Subcortical
71	Rectus_L	Gyrus rectus	REC.L	Prefontal
72	Rectus_R	Gyrus rectus	REC.R	Prefontal
73	Rolandic_Oper_L	Rolandic operculum	ROL.L	Frontal
74	Rolandic_Oper_R	Rolandic operculum	ROL.R	Frontal

75	Supp_Motor_Area_L	Supplementary motor area	SMA.L	Frontal
76	Supp_Motor_Area_R	Supplementary motor area	SMA.R	Frontal
77	SupraMarginal_L	Supramarginal gyrus	SMG.L	Parietal
78	SupraMarginal_R	Supramarginal gyrus	SMG.R	Parietal
79	Temporal_Inf_L	Inferior temporal gyrus	ITG.L	Temporal
80	Temporal_Inf_R	Inferior temporal gyrus	ITG.R	Temporal
81	Temporal_Mid_L	Middle temporal gyrus	MTG.L	Temporal
82	Temporal_Mid_R	Middle temporal gyrus	MTG.R	Temporal
83	Temporal_Pole_Mid_L	Temporal pole: middle temporal gyrus	TPOmid.L	Temporal
84	Temporal_Pole_Mid_R	Temporal pole: middle temporal gyrus	TPOmid.R	Temporal
85	Temporal_Pole_Sup_L	Temporal pole: superior temporal gyrus	TPOsup.L	Temporal
86	Temporal_Pole_Sup_R	Temporal pole: superior temporal gyrus	TPOsup.R	Temporal
87	Temporal_Sup_L	Superior temporal gyrus	STG.L	Temporal
88	Temporal_Sup_R	Superior temporal gyrus	STG.R	Temporal
89	Thalamus_L	Thalamus	THA.L	Subcortical
90	Thalamus_R	Thalamus	THA.R	Subcortical
91	Cerebelum_Crus1_L	Cerebelum-Crus1-L	lCbeCru1	Cerebellum_Superior
92	Cerebelum_Crus1_R	Cerebelum-Crus1-R.	rCbeCru1	Cerebellum_Superior
93	Cerebelum_Crus2_L	Cerebelum-Crus2-L	lCbeCru2	Cerebellum_Inferior
94	Cerebelum_Crus2_R	Cerebelum-Crus2-R	rCbeCru2	Cerebellum_Inferior
95	Cerebelum_3_L	Cerebelum-3-L	lCbe3	Cerebellum_Superior
96	Cerebelum_3_R	Cerebelum-3-R	rCbe3	Cerebellum_Superior
97	Cerebelum_4_5_L	Cerebelum-4-5-L	lCbe4-5	Cerebellum_Superior
98	Cerebelum_4_5_R	Cerebelum-4-5-R	rCbe4-5	Cerebellum_Superior
99	Cerebelum_6_L	Cerebelum-6-L	lCbe6	Cerebellum_Superior
100	Cerebelum_6_R	Cerebelum-6-R	rCbe6	Cerebellum_Superior
101	Cerebelum_7b_L	Cerebelum-7b-L	lCbe7b	Cerebellum_Inferior
102	Cerebelum_7b_R	Cerebelum-7b-R	rCbe7b	Cerebellum_Inferior
103	Cerebelum_8_L	Cerebelum-8-L	lCbe8	Cerebellum_Inferior
104	Cerebelum_8_R	Cerebelum-8-R	rCbe8	Cerebellum_Inferior
105	Cerebelum_9_L	Cerebelum-9-L	lCbe9	Cerebellum_Inferior
106	Cerebelum_9_R	Cerebelum-9-R	rCbe9	Cerebellum_Inferior
107	Cerebelum_10_L	Cerebelum-10-L	lCbe10	Cerebellum_Inferior
108	Cerebelum_10_R	Cerebelum-10-R	rCbe10	Cerebellum_Inferior
109	Vermis_1_2	Vermis-1-2	Ver1-2	Vermis
110	Vermis_3	Vermis-3	Ver3	Vermis
111	Vermis_4_5	Vermis-4-5	Ver4-5	Vermis
112	Vermis_6	Vermis-6	Ver6	Vermis
113	Vermis_7	Vermis-7	Ver7	Vermis

114	Vermis_8	Vermis-8	Ver8	Vermis
115	Vermis_9	Vermis-9	Ver9	Vermis
116	Vermis_10	Vermis-10	Ver10	Vermis
